# Failure of enhanced recovery after surgery in liver surgery: a systematic review and meta analysis

**DOI:** 10.3389/fmed.2023.1159960

**Published:** 2023-07-11

**Authors:** Qiuping Ren, Menghang Wu, Hong Yu Li, Jiafei Li, Zi Hang Zeng

**Affiliations:** ^1^Division of Liver Surgery, Department of General Surgery, West China Hospital, West China School of Nursing, Sichuan University, Chengdu, China; ^2^West China School of Medicine, West China Hospital, Sichuan University, Chengdu, Sichuan, China; ^3^Nursing Key Laboratory of Sichuan Province, West China Medical Center, Sichuan Medical University, Chengdu, China

**Keywords:** enhanced recovery after surgery, systematic review and meta-analysis, failure, liver surgery, meta analysis

## Abstract

**Purpose:**

This study aimed to conduct a systematic review of the literature to identify and summarize the existing evidence regarding ERAS failure and related risk factors after hepatic surgery. The objective was to provide physicians with a better understanding of these factors so that they can take appropriate action to minimize ERAS failure and improve patient outcomes.

**Method:**

A literature search of the PubMed MEDLINE, OVID, EMBASE, Cochrane Library, and Web of Science was performed. The search strategy involved terms related to ERAS, failure, and hepatectomy.

**Result:**

A meta-analysis was conducted on four studies encompassing a total of 1,535 patients, resulting in the identification of 20 risk factors associated with ERAS failure after hepatic surgery. Four of these risk factors were selected for pooling, including major resection, ASA classification of ≥3, advanced age, and male gender. Major resection and ASA ≥ 3 were identified as statistically significant factors of ERAS failure.

**Conclusion:**

The comprehensive literature review results indicated that the frequently identified risk factors for ERAS failure after hepatic surgery are linked to operative and anesthesia factors, including substantial resection and an American Society of Anesthesiologists score of 3 or higher. These insights will assist healthcare practitioners in taking prompt remedial measures. Nevertheless, there is a requirement for future high-quality randomized controlled trials with standardized evaluation frameworks for ERAS programs.

## Introduction

The utilization of liver resection as a definitive surgical intervention for a variety of malignant and benign conditions has been traditionally associated with high postoperative mortality and morbidity rates (1). However, with the technical progress of surgical personnel and standardization of surgical procedures, the mortality rate has decreased to an acceptable level of less than 5% (2). Despite this improvement, the rate of morbidity and complication remain high(30–40%), which not only delays discharge, causes suffering for the patient, and increases the risk of mortality, but also is associated with decreased overall long-term survival following surgery for malignant diseases (3–5). As such, the minimization of morbidity is crucial for improving outcomes.

There has been a shift in traditional postoperative treatments, with a focus on earlier mobilization and faster recovery after surgery over the past two decades (6). This has led to the development and implementation of Enhanced Recovery After Surgery (ERAS) programs, which have been extensively studied and shown to be safe and effective (7–9). In 2011, the ERAS Society published guidelines, further promoting the widespread adoption of these programs across various surgical fields (10, 11). Despite the demonstrated benefits of ERAS programs in reducing postoperative morbidity and length of hospital stay in patients undergoing hepatic surgery, several studies have reported that a subset of patients fail to fully adhere to or benefit from these protocols (12–14). To address this issue, it is crucial to identify and understand the risk factors associated with ERAS failure, and to develop strategies for early identification and intervention in those patients who are at risk of deviation from the established protocols. However, the literature on this topic is currently limited, as there is a lack of consensus on the definition of ERAS failure and the specific risk factors associated with it, particularly in the context of liver surgery.

The present study aimed to conduct a systematic review of the literature in order to identify and summarize existing evidence pertaining to the failure of ERAS protocols and associated risk factors among patients undergoing hepatic surgery. The utilization of this evidence would enable physicians to identify patients at risk of ERAS failure and subsequently implement timely interventions to address this issue.

## Materials and methods

### Search strategies

Original articles were searched for in databases including PubMed MEDLINE, OVID, EMBASE, Cochrane Library, and Web of Science until November 1, 2022. We used following terms for the literature search: (“enhanced recovery after surgery” OR ERAS OR “enhanced recovery program” OR ERP OR “enhanced recovery” OR fast-track) AND (failure OR fail) AND (hepatectomy OR “liver resection” OR “liver surgery”). The citation language was restricted to English.

### Inclusion and exclusion criteria

Articles were included if they evaluated any risk factor of ERAS failure in whether open or laparoscopic hepatectomy. Articles were excluded if they were: (1) prospective or retrospective studies but there were no control groups; (2) reviews, comments, conference abstracts, letters, and case reports; (3) animal experiments; and (4) only citations available, full texts not retrievable.

### Data extraction

Two reviewers independently reviewed all included articles, and extracted necessary data: author, publication year, country, research type, study population, definition of ERAS failure, and risk factors. Two reviewers independently evaluated the quality of evidence of included studies using the Newcastle-Ottawa Scale. If any disagreement was present, another reviewer was assigned to re-evaluate the study.

### Statistical analysis

Categorical data were synthesized as odds ratio (OR) with its 95% confidence interval (CI); continuous data measured with the same scale were synthesized using the weighted mean difference (WMD). If continuous data were demonstrated as medians and interquartile ranges (IQRs), the means and standard deviations (SDs) were estimated using an estimation method (15, 16). I-square (I2) was calculated to estimate heterogeneity. If I2 > 50%, the random effect model was used to integrate data, otherwise the fixed effect model was used. Begg’s test was used to evaluate publication bias. Signification level (α) was set to 0.05 for statistical tests.

## Results

### Study selection

**Figure 1:**
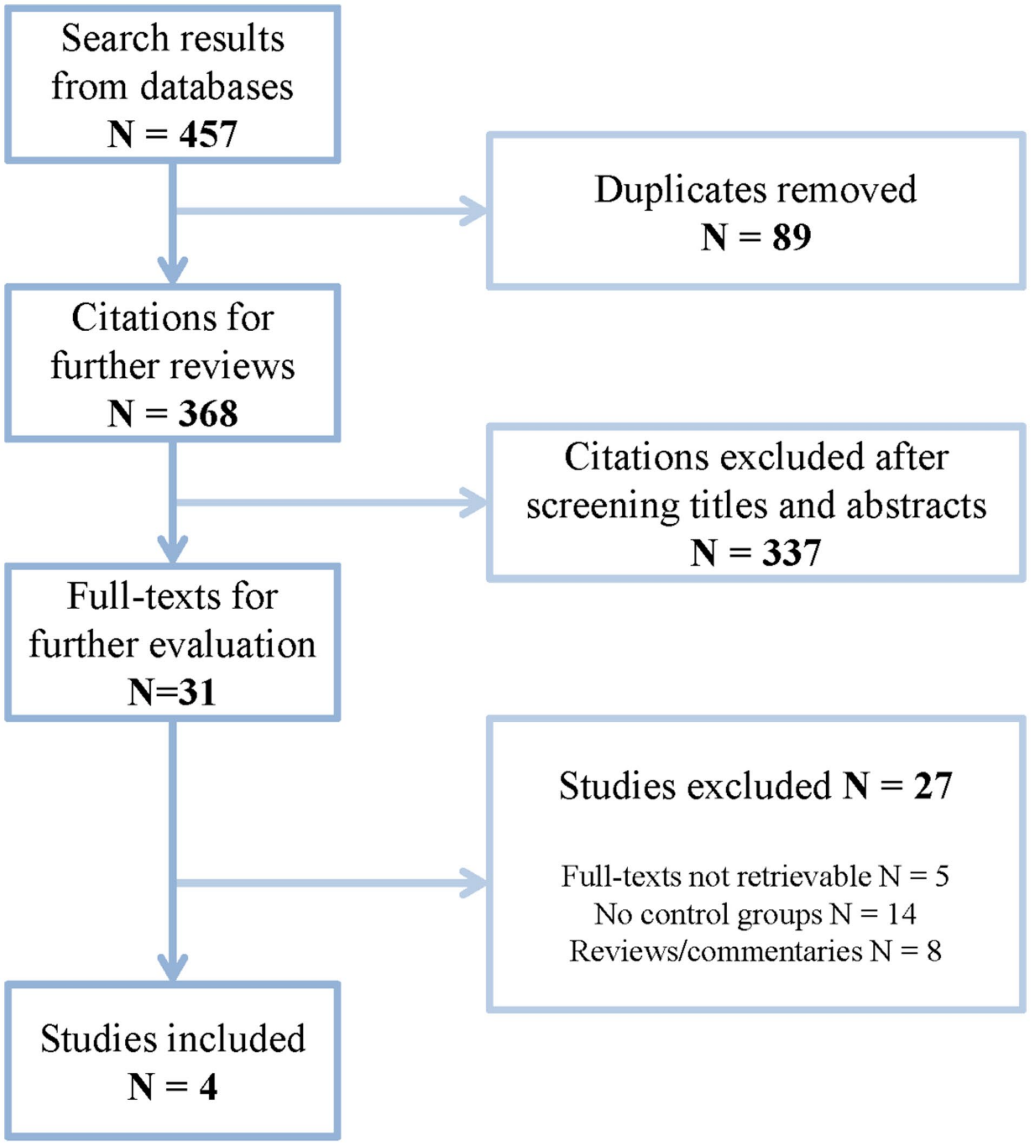
A flow chart of this study.

A flow chart shows the process of study inclusion in Figure 1. A total of 457 citations were found after initial literature search. After removing duplicates, there were 368 citations remaining for further review. After screening titles and abstracts, there were 31 articles remaining for further review. Finally, four studies were included in this systematic review and meta-analysis after reviewing full texts. The baseline information of these studies is shown in Table 1. All of them are retrospective studies and a total of 1,535 cases were involved in them.

**Table 1:** The baseline information of included studies.

Author	Year	Country	No. of patients	Nos. score	Definition of ERAS failure	Surgery type	Diagnosis	Follow up
Hughes	2016	UK	603	7	severe morbidity (Clavien Dindo grade≥3)	laparoscopic and open	HCC (*n* = 75) CLM (*n* = 381) other malignancy (*n* = 84) benign (*n* = 63)	30 days
Lee	2014	China	194	6	length of ICU stay more than 24 h after surgery unplanned admission to ICU within 3 0 days after surgery readmission to the hospital within 3 0 days after surgery reoperation for complications 30-day mortality	laparoscopic and open	NA	30 days
Takamoto	2014	Japan	200	6	On postoperative day 6, if following criteria were not met: normal or decreasing serum bilirubin level absence of fever (<37.5C for >48 h) adequate pain control with oral analgesics only ability to consume water and solid foods without requiring intravenous fluids adequate mobilization independently or at the preoperative level	open	HCC (*n* = 55) metastases (*n* = 85) other malignancy (*n* = 60) benign (*n* = 14)	6 days
Wong-Lun-Hing	2017	Netherland	53	7	severe morbidity (Clavien Dindo grade≥3a)	laparoscopic and open	HCC (*n* = 24) metastases (*n* = 421) other malignancy (*n* = 41) benign (*n* = 47) other (*n* = 5)	90 days

### ERAS elements

The ERAS protocol is summarized in Table 2. The ERAS items applied at least four times included education and intake of liquid carbohydrates in the preoperative period, minimal invasive surgery in the intraoperative period, and no nasogastric tube use and oral food intake within 24 h after surgery in the postoperative period. Immediate mobilization and removal of the urinary catheter within 24 h after surgery were reported in all included studies.

**Table 2:** Perioperative care interventions of included studies.

	Lee et al. (17)	Hughes et al. (18)	Takamoto et al. (19)	Wong-Lun-Hing et al. (20)
Preoperative				
Education and counseling	√	√	√	
No prolonged starvation	√	√	√	
No bowel preparation		√	√	
Carbohydrate drink				
One-shot prophylactic antibiotics	√	√		√
Prevention of ileus				
Intraoperative				
Short-acting anesthetics		√		√
Fluid restriction		√	√	
Epidural anesthesia	√	√	√	
TAP/local wound analgesia				
Minimal invasive surgery				√
Keep patient warm	√	√	√	√
Postoperative				
No NG tube	√	√		√
Immediate mobilization	√	√	√	
Multimodal opioid-sparing analgesia		√		
Begin oral food intake on POD 1	√	√		√
Removal of urinary catheter on POD 1				√
Regular laxatives				
Discontinuation of IVF		√	√	

TAP, transversus abdominis plane, NG, nasogastric, POD, postoperative day, IVF, intravenous fluid.

### Risk factors

**Figure 2:**
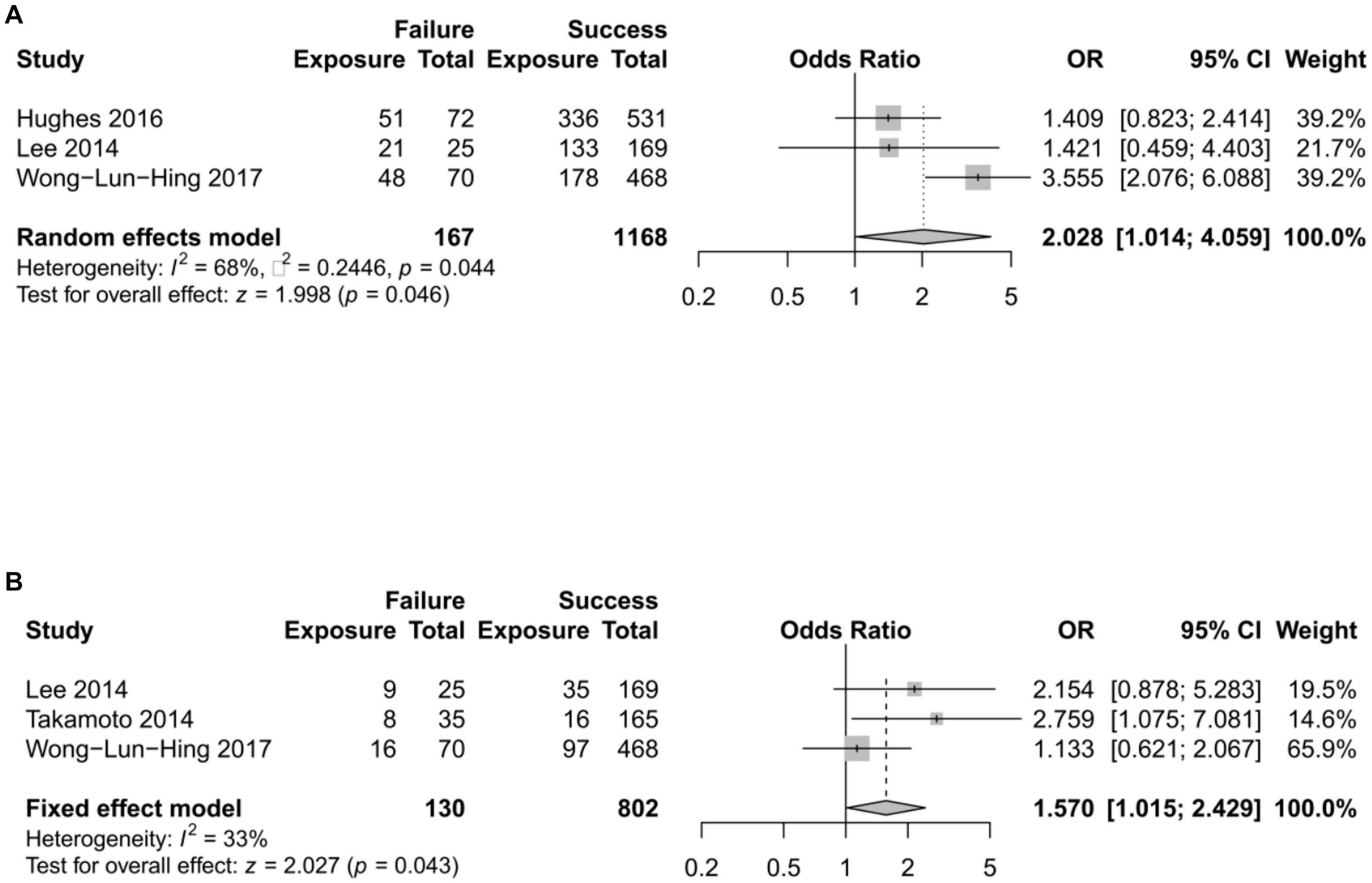
The effects of significant factors on ERAS failure. The horizontal lines with centered points and shaded squares stand for each study, while the diamond stand for pooled effects. **(A)** The effect of major resection. **(B)** The effect of ASA ≥3. OR, odds ratio; CI, confidence interval.

**Figure 3:**
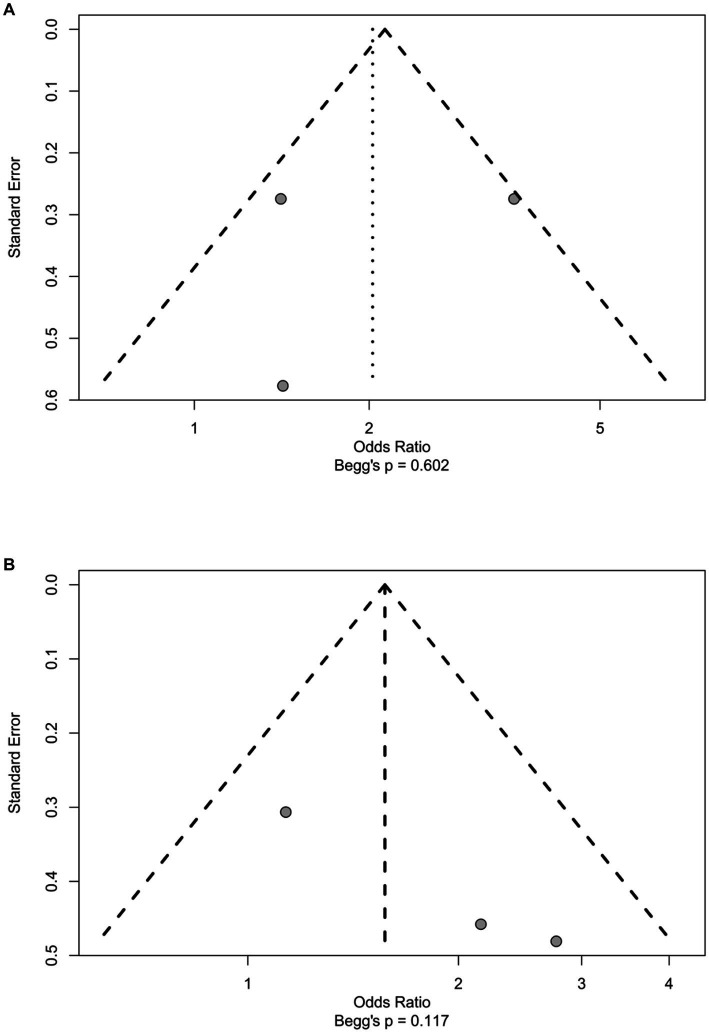
The funnel plot of significant factors. The distribution of the points and Begg’s *p* value suggest that there were no obvious sign of publicayion bias. **(A)** Major resection. **(B)** ASA ≥3.

A total of 20 factors were involved in these four studies (Table 3). Among them, four risk factors could be pooled for the meta-analysis. Major resection and ASA ≥ 3 were identified as statistically significant factors of ERAS failure (*p* = 0.048 and *p* = 0.043 respectively; Figure 2). A funnel plot shows there is no obvious publication bias for both factors (Begg’s *p* = 0.602 and Begg’s *p* = 0.117; Figure 3). Older age and male gender were risk factors of ERAS failure but were not significant (Supplementary Figure 1).

**Table 3:** Risk factors of included studies.

Factor	Note	No. of studies that reported this factor	Meta-analysis	Risk	95% CI	*p*
Age	Years	3	Yes	WMD = 1.9	(−1.2, 5.8)	0.229
Gender	Male/female	4	Yes	OR = 1.258	(0.922, 1.717)	0.148
ASA ≥ 3		3	Yes	OR = 1.570	(1.015, 2.429)	**0.043**
Major resection		3	Yes	OR = 2.028	(1.014, 4.059)	**0.046**
Extended resection		1	No	OR = 4.079	(2. 177, 7.642)	**<** **0.0001**
Current smoker		1	No	RR = 2.21	(1. 10, 4.46)	**0.027**
High ALT/GPT	More than 67 IU/L in men and more than 55 IU/L in women	1	No	RR = 3.55	(1.68, 7.49)	**0.001**
Blood loss during operation	Grams	2	No	WMD = 505.0	(354.2, 655.9)	**<** **0.0001**
Blood transfusion		2	No	OR = 2.042	(0.429, 9.719)	0.370
Operation time	Minutes	2	No	WMD = 89.3	(−13. 1, 191.7)	0.087
Chemotherapy		2	No	OR = 1.182	(0.814, 1.715)	0.879
BMI		1	No	OR = 0.997	(0.930, 1.068)	0.924
Low albumin	≤30 g/L	1	No	OR = 2.420	(0.989, 5.922)	0.053
High bilirubin	≥20 μmol/L	1	No	OR = 1.850	(0.931, 3.676)	0.079
Pringle maneuvre		1	No	OR = 1.559	(0.835, 2.911)	0.163
Caudate lobe resection		1	No	OR = 0.569	(0. 131, 2.468)	0.759
Repeat hepatectomy		1	No	OR = 1.439	(0.716, 3. 111)	0.285
Central resection		1	No	OR = 1.352	(0.381,4.795)	0.640
Thoracotomy		1	No	OR = 1.27	(0.37,5.91)	0.721
Hepaticojejunostomy		1	No	OR = 1.34	(0.45,3.97)	0.596

More blood loss during operation was a statistically significant risk factor whether pooled or not (Supplementary Figure 1). However, only two studies reported this variable, therefore it was not eligible for the meta-analysis and was considered as a factor identified from individual studies. The effects of peration time, blood loss during surgery, blood transfusion and the use of chemotherapy were also pooled, but their effects were not significant either (Supplementary Figure 1).

Other factors identified from individual studies were mostly not significant. Nevertheless, there were two factors reported in individual studies. Lee et al. (17) reported that high ALT/GPT and smoking were independent risk factors of ERAS failure (RR = 3.55, *p* = 0.001 and RR = 2.21, *p* = 0.027, respectively), where high ALT/GPT was defined as more than 67 IU/L in men and more than 55 IU/L in women. Hughes et al. (18) reported that extended resection was an independent risk factor of ERAS failure (OR = 4.079, *p* < 0.0001).

## Discussion

The present investigation conducted a systematic review of the literature to identify four articles that pertained to the failure of ERAS protocols and associated risk factors in patients who underwent hepatic surgery. The results of this study may be useful in identifying patients who are at a higher risk of ERAS failure, thereby allowing for timely implementation of corrective measures. Due to the substantial heterogeneity in study design and variations in ERAS protocols among the included studies, the meta-analysis was only performed for four risk factors. As far as we are aware, this is the initial systematic review and meta-analysis that specifically examines the risk factors for ERAS failure in the context of hepatic surgery.

The utilization of ERAS protocols has gained significant traction in the field of surgical operations, with numerous studies demonstrating the effectiveness of these protocols in reducing hospital admission times, morbidity rates and overall improving patient outcomes. This has led to the widespread acceptance of ERAS as a standard of care in surgeries (12, 13, 17, 18). Similarly, there has been a growing interest in the application of ERAS protocols in the field of liver resectional surgery. Recent studies, including several randomized controlled trials, have demonstrated the safety and feasibility of ERAS in liver surgery, as well as a reduction in morbidity rates following liver resections (19, 20). Systematic reviews of observational studies have demonstrated the safety and feasibility of ERAS programs in hepatobiliary surgery (18, 21, 22). Such programs, when compared to traditional clinical pathways, have been found to have similar risks of readmission, morbidity, and mortality, and have been associated with reduced duration of postoperative length of stay and overall hospital costs. However, the degree of compliance with core components of enhanced recovery after liver surgery programs among high-volume European centers has been found to vary (23).

In past few years, a number of high-volume medical centers have reported achieving approximately a zero-mortality rate after liver resection, suggesting that the surgical procedure and perioperative care for patients undergoing this procedure have a high rate of successful completion (23). However, it is important to note that the maintenance of a low rate of mortality for liver resection protocols cannot be guaranteed, even with significant improvements in perioperative care. Although the standard ERAS protocol guidelines recommend 26 perioperative care interventions, not all of those past interventions were implemented in the studies that were included in the review (24). The implementation of an ERAS for an invasive hepatectomy procedure should be carefully evaluated, taking into consideration not only reductions in length of hospital stay and medical expenses, but also potential risks to the patient. The literature has reported a wide range of morbidity rates following liver surgery, with estimates ranging from 22 to 45% (1, 22, 25). When comparing these rates to those of liver resection performed under ERAS protocols, the reported morbidity ranges from 11 to 46% (14). Overall, it appears that the use of ERAS principles in the management of liver resection may result in a reduction in morbidity compared to traditional practices. However, it should be noted that the literature does not indicate any significant impact of ERAS care on specific surgical morbidity.

Recent studies have demonstrated a trend towards increasingly favorable long-term outcomes following major resection for advanced disease (2–4, 25, 26). This trend serves as justification for an aggressive surgical approach in these cases. However, our study suggested that major resection was identified as statistically significant factors of ERAS failure. The current study observed significantly elevated rates of liver impairment following extended resections in comparison to major and minor hepatic resections (27). Despite the utilization of advanced techniques, such as Partial Volume Resection (PVE), tumor volume reduction, and two-stage procedures, to mitigate the risk of liver failure, this complication remains a concern in the context of major resections. Furthermore, the study observed significantly higher rates of bile leak and abscess formation after extended resections compared to non-extended resections (27, 28). Despite the implementation of these techniques, hepatic insufficiency remains a complication that requires ongoing attention.

In addition to surgical challenges, major resections also present difficulties in addressing non-surgical complications and management. The analgesic regimens after surgery are still complex, and the metabolism of different analgesic drugs is currently not well understood following major resection (29). The American Society of Anesthesiologists (ASA) guidelines from 2016 stipulate that ERAS is an integral component of the Perioperative Surgical Home (PSH) and recommends the implementation of a multimodal, opioid-sparing approach for the management of postoperative pain (30–32). In line with this recommendation, dexmedetomidine is increasingly being utilized as part of ERAS protocols in conjunction with regional nerve blocks and other medications, in order to achieve satisfactory postoperative outcomes while minimizing opioid consumption (33, 34). Furthermore, postoperative nutrition is of paramount importance in the context of liver resection due to the increased surgical insult and risk of sepsis. As such, further research is necessary to investigate the effects of small liver remnant volume on outcomes following liver resection and to determine how ERAS protocols should be adapted to account for the unique aspects of liver surgery that cannot directly draw lessons from other general abdominal surgery.

Our review had several limitations that should be acknowledged. Firstly, all of the studies included in the review employed a retrospective cohort design, which is prone to information bias and publication bias, as authors are more likely to submit studies with positive results for publication. Secondly, the heterogeneity of the ERAS protocols utilized in the included studies precluded a comprehensive assessment of all 26 elements recommended in the guidelines, and the level of compliance with these elements was not reported. To address this limitation, future studies with standardized ERAS protocols and detailed information on compliance with all recommended elements would be beneficial. Additionally, the scope of the review was limited to studies that reported on ERAS failure, which resulted in a small sample size and precluded an examination of other outcomes such as delayed discharge and high-quality randomized controlled trials (RCTs) that follow a standardized framework for evaluating ERAS programs should be conducted in the future might address this limitation.

## Conclusion

The results of a comprehensive literature review indicated that the most commonly reported risk factors for ERAS failure after hepatic surgery include operative and anesthetic factors such as major resection and ASA ≥ 3. These findings will aid healthcare providers in taking corrective actions promptly. However, there is a need for high-quality randomized controlled trials with standardized evaluation frameworks for ERAS programs in the future.

## Author contributions

QR and JL had full access to all the data in the study and take responsibility for the integrity of the data and the accuracy of the data analysis. QR, MW, HL, JL, and ZZ: concept and design and drafting of the manuscript. QR, HL, JL, and ZZ: acquisition, analysis, or interpretation of data. QR, MW, HL, and JL: critical revision of the manuscript for important intellectual content. QR, MW, and JL: supervision. All authors contributed to the article and approved the submitted version.

## Funding

The name of granting agencies: West China Nursing Discipline Development Special fund, Sichuan University grant number: HXHL20043. A short description: analysis of the elements that lead to failure and development of a model for predicting the likelihood of quick rehabilitation surgery following partial hepatectomy.

## Conflict of interest

The authors declare that the research was conducted in the absence of any commercial or financial relationships that could be construed as a potential conflict of interest.

## Publisher’s note

All claims expressed in this article are solely those of the authors and do not necessarily represent those of their affiliated organizations, or those of the publisher, the editors and the reviewers. Any product that may be evaluated in this article, or claim that may be made by its manufacturer, is not guaranteed or endorsed by the publisher.
