# Supplementary figures and images for: Failure of enhanced recovery after surgery in liver surgery: a systematic review and meta analysis
Source: Front Med (Lausanne). 2023 Jul 11;10:1159960. doi: 10.3389/fmed.2023.1159960 (PMC10366385; doi:10.3389/fmed.2023.1159960)

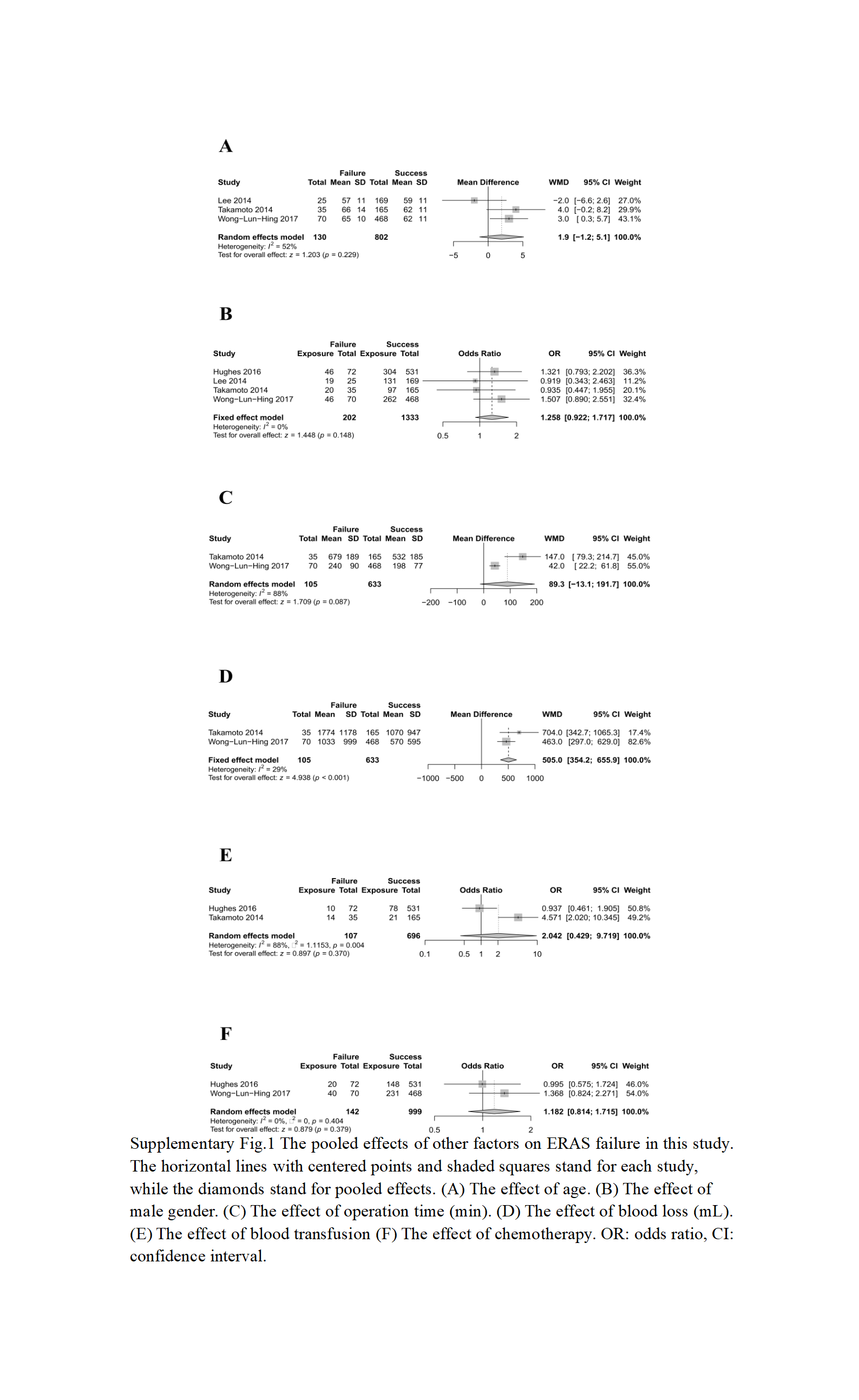

Supplement: Supplementary file 1 [file Image_1.tif]
